# A Characterization of Optimal Prefix Codes

**DOI:** 10.3390/e26121000

**Published:** 2024-11-21

**Authors:** Spencer Congero, Kenneth Zeger

**Affiliations:** Department of Electrical and Computer Engineering, University of California, La Jolla, San Diego, CA 92093-0407, USA; scongero@ucsd.edu

**Keywords:** prefix codes, Kraft inequality, Huffman codes, unique decodability

## Abstract

A property of prefix codes called strong monotonicity is introduced, and it is proven that for a given source, a prefix code is optimal if and only if it is complete and strongly monotone.

## 1. Introduction

This paper concerns variable-length binary codes used to transmit or store source symbols generated by a finite probability distribution. Our main result is the following characterization of binary prefix codes that achieve the minimal possible average codeword length for a given probability distribution over a finite symbol set.

**Theorem** **1.** *A prefix code is optimal if and only if it is complete and strongly monotone.*

In the remainder of the paper, we first define the terminology, then give historical background, and finally prove the main result.

An *alphabet* is a finite set *S*, and a *source* with alphabet *S* is a random variable *X* that takes on each value y∈S with probability P(y). The probability of any subset B⊆S is denoted by $P\left(B\right)=\sum_{y\in B}P\left(y\right)$.

A *code* for source *X* is a mapping $C:S⟶{\left\{0,1\right\}}^{*}$, and for each y∈S, the binary string C(y) is a *codeword* of *C*. A *prefix code* is a code where no codeword is a prefix of any other codeword.

A *code tree* for a prefix code *C* is a rooted binary tree whose leaves correspond to the codewords of *C*. By convention, each edge leading to a left child will be labeled 0 and each edge leading to a right child will be labeled 1. The codeword associated with each leaf is the binary word describing the path from the root to the leaf. The *r*th *row* of a code tree is the set of nodes whose path length from the root is *r*, and we will view a code tree’s root as being on the top of the tree with the tree growing downward. That is, row *r* of a code tree is “higher” in the tree than row r+1. If *x* and *y* are nodes in a code tree, then *x* is a *descendant* of *y* if there is an upward path of length zero or more from *x* to *y*. Two nodes in a tree are called *siblings* if they have the same parent. In a code tree, the probability of a leaf is the probability of its codeword, and therefore also the corresponding source symbol. For any collection *A* of nodes in a code tree, let P(A) denote the probability of the set of all leaf descendants of *A* in the tree.

A (binary) *Huffman tree* is a code tree constructed from a source by recursively merging two smallest-probability nodes until only one node with probability 1 remains. (For more details about Huffman codes, see Section 5.6 of [1].) The leaf nodes in the tree correspond to initial source probabilities. A *Huffman code* for a given source is a mapping of source symbols to binary words by assigning the source symbol of each leaf in the Huffman tree to that leaf’s codeword.

Given a source with alphabet *S* and a prefix code *C*, for each y∈S, the length of the binary codeword C(y) is denoted $l_{C}\left(y\right)$. Two codes $C_{1}$ and $C_{2}$ are *length-equivalent* if $l_{C_{1}}\left(y\right)=l_{C_{2}}\left(y\right)$ for every source symbol y∈S. The *average length* of a code *C* for a source with alphabet *S* is $\sum_{y\in S}l_{C}\left(y\right)P\left(y\right)$. A prefix code is *optimal* for a given source if no other prefix code achieves a smaller average codeword length for the source.

The *Kraft sum* [2] of a sequence of non-negative integers $l_{1},\cdots ,l_{k}$ is $2^{-l_{1}}+\cdots +2^{-l_{k}}$. We extend the definition of “Kraft sum” to sets of source symbols with respect to a code as follows. If *C* is a prefix code for a source with alphabet *S*, and U⊆S, then the Kraft sum of *U* is

$$\begin{array}{cc} K_{C}\left(U\right) & =\sum_{x\in U}2^{-l_{C}\left(x\right)}. \end{array}$$

The following lemma is a standard result in most information theory textbooks and is used in the proofs of Lemma 4 and Theorem 1.

**Lemma** **1** (Kraft Inequality converse ([1] Theorem 5.2.1))**.** *If a sequence $l_{1},\cdots ,l_{n}$ of positive integers satisfies $2^{-l_{1}}+\cdots +2^{-l_{n}}\leq 1$, then there exists a binary prefix code whose codeword lengths are $l_{1},\cdots ,l_{n}$.*

A code is *complete* if every non-root node in its code tree has a sibling, or, equivalently, if every node has either zero or two children. (Our usage of the word “complete” has also been referred to in the literature as “full”, “extended”, “saturated”, “exhaustive”, and “maximal”.) A code *C* for a given source is *monotone* if, for any source symbols u,v∈S, whenever $l_{C}\left(u\right)<l_{C}\left(v\right)$, we have P(u)≥P(v). Completeness and monotonicity are necessary for optimality, as stated in the following lemma.

**Lemma** **2** (Huffman [3] p. 1099)**.** *For any source, if a prefix code is optimal, then it is complete and monotone.*

In this paper, we provide a necessary and sufficient characterization of optimal prefix codes by introducing a new criterion called “strong monotonicity”.

**Definition** **1.** *Given a source with alphabet S, a prefix code C is strongly monotone if, for any A,B⊆S, whenever $K_{C}\left(A\right)=2^{-i}>2^{-j}=K_{C}\left(B\right)$ for some integers i and j, we have P(A)≥P(B).*

Monotonicity can be viewed as a special case of strong monotonicity when the subsets *A* and *B* are restricted to be collections of leaf descendants of individual tree nodes.

## 2. Prior Work

Huffman codes [3] were invented in 1952 and are used today in many practical data compression applications, such as for text, audio, image, and video coding, and are known to be optimal [1].

For a given source, Huffman codes and their corresponding code trees are not generally unique, due to three types of choices that arise during the tree construction that can be decided arbitrarily: (i) When two nodes are merged, the choice of which node becomes a left child and which becomes a right child is arbitrary; (ii) If there are three or more smallest-probability nodes, then which two of them to merge is arbitrary; (iii) If there is a unique smallest-probability node and two or more second-smallest-probability nodes, then which of these to merge with the smallest-probability node is arbitrary. These latter two cases do not occur if probability “ties” are absent among tree nodes, which is almost surely true if the source itself is randomly chosen from a continuous distribution.

For many applications, the average length of a prefix code is the primary concern, while in other applications, the specific binary codewords included in an optimal code may also be critical, such as for reducing average resynchronization time when channel errors can occur (e.g., [4,5,6,7,8,9,10,11]).

In addition to a given source having multiple Huffman codes, the source can also have multiple non-Huffman codes that achieve the same minimal average codeword length as Huffman codes. Furthermore, even the topologies of the code trees for optimal non-Huffman codes can be different.

Both algorithmic and mathematical characterizations of Huffman codes, and more generally optimal prefix codes, have been of great interest over the last 70 years.

Algorithmically, certain equivalences between codes have been described in the literature in terms of various “node swap” transformations of the corresponding code trees. “Same-parent” node swaps consist of switching the two siblings (and the entire subtrees hanging from them) of a parent node in the tree. Similarly, “same-row” node swaps switch two nodes in the same tree row, and “same-probability” node swaps switch two tree nodes having the same probability. Any two complete prefix codes that are length-equivalent can be obtained from each other by a series of same-row node swaps. Also, any two Huffman codes for the same source can be obtained from each other by a series of same-parent and same-probability node swaps [8]. Additionally, since every optimal code is length-equivalent to some Huffman code [12], any two optimal codes can be obtained from each other by a series of same-row and same-probability node swaps.

In 1978, Gallager [13] gave a useful non-algorithmic characterization of Huffman codes (stated below in Lemma 3) as those prefix codes possessing a “sibling property”.

**Definition** **2.** *A binary code tree has the sibling property if it is complete and if the nodes can be listed in order of non-increasing probability with each node being adjacent in the list to its sibling.*

The next lemma is used in the proof of Theorem 1.

**Lemma** **3** (Gallager [13]’s Theorem 1)**.** *For any source, a prefix code is a Huffman code if and only if its code tree has the sibling property.*

For the broader class of optimal prefix codes, no characterization analogous to the sibling property has been previously given. Only the sufficient condition given by the sibling property has been known. One known necessary (but not sufficient, even also assuming completeness) condition for a prefix code to be optimal is “monotonicity”, which states generally that code tree node probabilities decrease moving downward in the code tree.

Theorem 1 provides both a necessary and sufficient condition for optimality. Specifically, for a given source, a prefix code is optimal if and only if it is complete and strongly monotone. Another recent work [14] uses Theorem 1 to prove results about the competitive optimality of Huffman codes.

**Figure 1:**
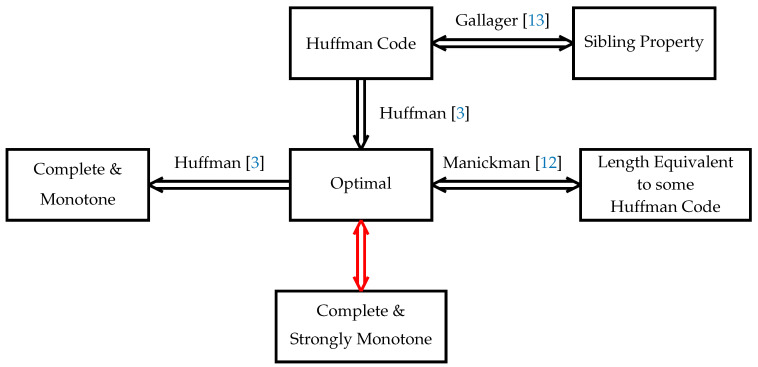
Logical implications of prefix code properties for a given source [3,12,13]. The red arrows indicate new results presented in this paper.

Figure 1 depicts the main result relating to Theorem 1, along with some known prior art.

## 3. Characterization of Optimal Prefix Codes

In this section, we prove Theorem 1, which gives a new characterization of optimal prefix codes for a given source.

While all Huffman codes are optimal and were characterized by Gallager in terms of the sibling property, not all optimal codes are Huffman codes. Theorem 1 shows that a prefix code is optimal if and only if it is complete and strongly monotone. The combination of completeness and strong monotonicity is weaker than the sibling property, and thus a broader class of prefix codes (namely, the optimal ones) satisfies this combination.

The strongly monotone property reduces to Gallager’s monotone property when the set *A* consists of all leaf descendants of a single tree node, and *B* consists of all leaf descendants of a different tree node. Example 1 illustrates that these two properties are not equivalent. Specifically, the example shows that prefix code *C* is not strongly monotone because $K_{C}\left(\left\{c,d\right\}\right)=2^{-1}>2^{-2}=K_{C}\left(\left\{a\right\}\right)$ but $P\left(\left\{c,d\right\}\right)=\frac{1}{4}<\frac{3}{8}=P\left(\left\{a\right\}\right)$.

Completeness and monotonicity do not imply strong monotonicity, nor do they imply optimality, as illustrated in the following example.

**Figure 2:**
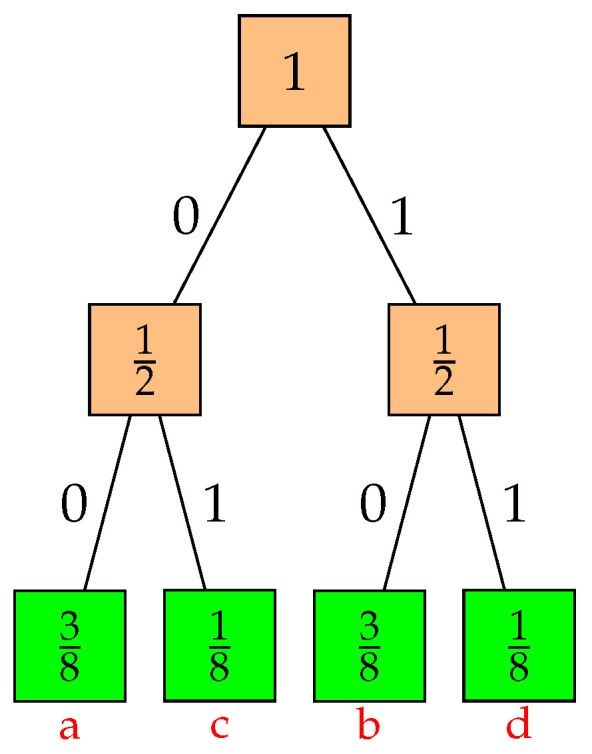
A code tree illustrating monotonicity without strong monotonicity.

**Example** **1** (Complete and monotone ⇏ optimal)**.** *See Figure 2. A balanced prefix code C for a source with symbols a, b, c, and d, and probabilities $\frac{3}{8},\frac{3}{8},\frac{1}{8},\;and\;\frac{1}{8}$, respectively, is complete, monotone, and has an average length of 2. But C is not optimal since a Huffman code has codewords of lengths 1,2,3, and 3 and a smaller average length of 15/8.*

Strong monotonicity of a prefix code does not imply that the code is complete, and hence the code may not be optimal, as illustrated in the following simple example.

**Figure 3:**
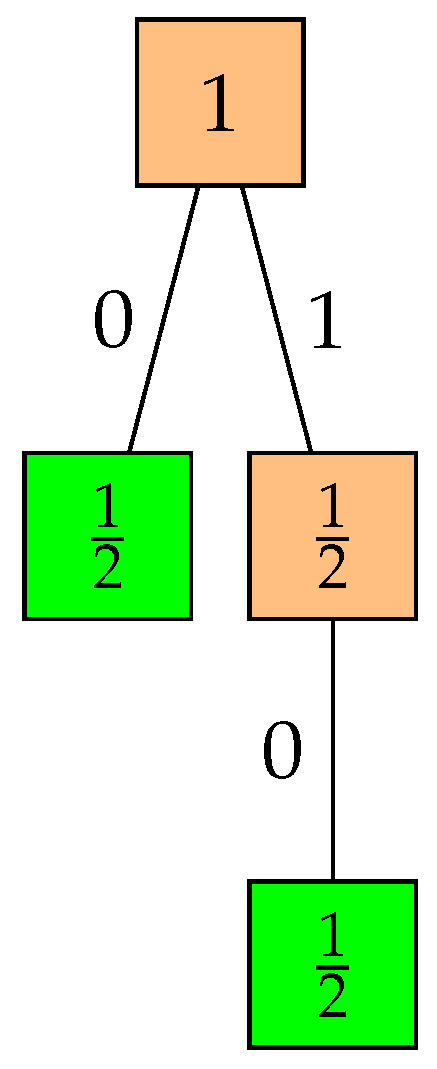
A non-complete prefix code tree illustrating strong monotonicity without optimality.

**Example** **2** (Strongly monotone ⇏ optimal)**.** *See Figure 3.*

The following lemma easily follows from the proof of Lemma 1. This lemma relies on our defining assumption that sources (and thus codes) are finite. Prefix codes for infinite sources need not satisfy the lemma below.

**Lemma** **4.** *A prefix code is complete if and only if for every node u in its code tree, the Kraft sum of the set of leaf descendants of u equals $2^{-i}$, where u lies in the ith row of the code tree. Also, a prefix code is complete if and only if its Kraft sum equals* 1.

The following lemma lists properties that do not change among length-equivalent prefix codes.

**Lemma** **5.** *If two prefix codes are length-equivalent, then each of the following properties holds for one code if and only if it holds for the other code: (i) completeness; (ii) strong monotonicity; and (iii) optimality.*

**Proof.**  Let *S* be the source alphabet. Let *C* and $C^{\prime }$ be length-equivalent prefix codes, i.e., $l_{C}\left(y\right)=l_{C^{\prime }}\left(y\right)$ for all y∈S. Then, for all y∈S,

$$\begin{array}{cc} K_{C}\left(\left\{y\right\}\right)\; & =2^{-l_{C}\left(y\right)}=2^{-l_{C^{\prime }}\left(y\right)}=K_{C^{\prime }}\left(\left\{y\right\}\right). \end{array}$$

Since

$$\begin{array}{cc} \sum_{y\in S}K_{C}\left(\left\{y\right\}\right)\; & =\sum_{y\in S}K_{C^{\prime }}\left(\left\{y\right\}\right), \end{array}$$

$K_{C}\left(S\right)=1$ if and only if $K_{C^{\prime }}\left(S\right)=1$, so Lemma 4 implies that *C* is complete if and only if $C^{\prime }$ is complete.

Suppose *C* is strongly monotone. Let A,B⊆S with $K_{C^{\prime }}\left(A\right)=2^{-i}$ and $K_{C^{\prime }}\left(B\right)=2^{-j}$ for some integers i,j such that 0≤i<j. Since $K_{C}\left(A\right)=K_{C^{\prime }}\left(A\right)=2^{-i}$ and $K_{C}\left(B\right)=K_{C^{\prime }}\left(B\right)=2^{-j}$, we have P(A)≥P(B) since *C* is strongly monotone. Thus, $C^{\prime }$ is also strongly monotone.

Let *X* be a source random variable. The average length of code *C* is

$$\begin{array}{cc} E[l_{C}\left(X\right)]\; & =\sum_{y\in S}P\left(y\right)l_{C}\left(y\right)=\sum_{y\in S}P\left(y\right)l_{C^{\prime }}\left(y\right)=E\left[l_{C^{\prime }}\left(X\right)\right], \end{array}$$

so *C* is optimal if and only if $C^{\prime }$ is optimal. □

The following proves our main result.

**Proof** **of** **Theorem** **1.** Let *S* be an alphabet and let *X* be a source on *S*.

First, suppose *C* is an optimal prefix code for *X*. Then, *C* is complete by Lemma 2. Suppose for contradiction that *C* is not strongly monotone. Then, there exist subsets A,B⊆S such that $K_{C}\left(A\right)=2^{-i}>2^{-j}=K_{C}\left(B\right)$ for some integers *i* and *j*, but P(A)<P(B). Define a new prefix code $C^{\prime }$ such that for all u∈S,

$$\begin{array}{cc} l_{C^{\prime }}\left(u\right)\; & =l_{C}\left(u\right)+\left\{\begin{array}{cc} j-i & \mathrm{if}\;u\in A-B\\ i-j & \mathrm{if}\;u\in B-A\\ 0 & \mathrm{otherwise} \end{array}\right. \end{array}$$

Note that such a prefix code $C^{\prime }$ exists by Lemma 1, since $K_{C}\left(S\right)\leq 1$ and

$$\begin{array}{cc} K_{C^{\prime }}\left(S\right)-K_{C}\left(S\right)\; & =\sum_{u\in A-B}\left(2^{-l_{C^{\prime }\left(u\right)}}-2^{-l_{C(u)}}\right)+\sum_{u\in B-A}\left(2^{-l_{C^{\prime }\left(u\right)}}-2^{-l_{C(u)}}\right)\\ \; & =\sum_{u\in A-B}2^{-l_{C(u)}}\left(2^{l_{C(u)}-l_{C^{\prime }\left(u\right)}}-1\right)+\sum_{u\in B-A}2^{-l_{C(u)}}\left(2^{l_{C(u)}-l_{C^{\prime }\left(u\right)}}-1\right)\\ \; & =\left(2^{-(j-i)}-1\right)K_{C}\left(A-B\right)+\left(2^{-(i-j)}-1\right)K_{C}\left(B-A\right)\\ \; & <\left(2^{-(j-i)}-1\right)K_{C}\left(A-B\right)+\left(2^{-(i-j)}-1\right)K_{C}\left(B-A\right)\\ \; & \;\;\;\;\;\;\;\;+\left(2^{-(j-i)}+2^{-(i-j)}-2\right)K_{C}\left(A\cap B\right)\\ \; & =\left(2^{-(j-i)}-1\right)K_{C}\left(A\right)+\left(2^{-(i-j)}-1\right)K_{C}\left(B\right)\\ \; & =\left(2^{-(j-i)}-1\right)2^{-i}+\left(2^{-(i-j)}-1\right)2^{-j}\\ \; & =0, \end{array}$$

where the inequality above follows since *i* and *j* are non-negative integers satisfying j>i, implying $2^{-(i-j)}\geq 2$ and $2^{-(j-i)}>0$. But

$$\begin{array}{cc} E\left[l_{C^{\prime }}\left(X\right)\right]-E\left[l_{C}\left(X\right)\right]\; & =\sum_{u\in A-B}P\left(u\right)\left(l_{C^{\prime }\left(u\right)}-l_{C(u)}\right)+\sum_{u\in B-A}P\left(u\right)\left(l_{C^{\prime }\left(u\right)}-l_{C(u)}\right)\\ \; & =(j-i)P(A-B)+(i-j)P(B-A)\\ \; & =(j-i)(P(A)-P(B))\\ \; & <0, \end{array}$$

which contradicts the optimality of *C*. Thus, *C* is strongly monotone.

Now, suppose *C* is complete and strongly monotone, and let *T* be the code tree for *C*. The completeness of *T* implies that every row of *T* below the root has an even number of nodes since each node has a sibling. The following iterative procedure constructs a code tree $T^{\prime }$ that is length-equivalent to *T* and whose node probabilities are non-increasing from left to right in each row. Begin by listing the leaves on the bottom row of *T* in order of non-increasing probability and combining them as siblings in pairs; this is possible since there are an even number of such leaves on the row. Then, list the parent nodes just created and the leaves in the second-lowest row of *T* in order of non-increasing probability and combine them as siblings in pairs; again, this is possible for the same reason as in the previous step. Continue this procedure from the bottom row to the top row, until $T^{\prime }$ is constructed. The construction of $T^{\prime }$ preserves which row its leaves came from in *T*, so $T^{\prime }$ is length-equivalent to *T*.

Let *u* be a node in row *i* of $T^{\prime }$ and let *v* be a node in row *j* of $T^{\prime }$, where j>i. Let $C^{\prime }$ be the prefix code whose code tree is $T^{\prime }$, and let U,V⊆S be the sets of source symbols corresponding to the leaf descendants of *u* and *v*, respectively. Since *C* is complete, Lemma 5 implies $C^{\prime }$ is complete. Lemma 4 then implies $K_{C^{\prime }}\left(U\right)=2^{-i}$ and $K_{C^{\prime }}\left(V\right)=2^{-j}$, and since $C^{\prime }$ is length-equivalent to *C*, we have

$$\begin{array}{c} K_{C}\left(U\right)=K_{C^{\prime }}\left(U\right)=2^{-i}>2^{-j}=K_{C^{\prime }}\left(V\right)=K_{C}\left(V\right). \end{array}$$

since *C* is strongly monotone, P(u)=P(U)≥P(V)=P(v). Therefore, the list of nodes of $T^{\prime }$ in raster-scan order, beginning at the root node and moving down row-by-row, left-to-right in each row, has each node appearing adjacent to its sibling and the node probabilities non-increasing. Since in addition $C^{\prime }$ is complete, $C^{\prime }$ satisfies the sibling property, and so Lemma 3 implies that $C^{\prime }$ is a Huffman code. Thus, $C^{\prime }$ is optimal, and since *C* is length-equivalent to $C^{\prime }$, Lemma 5 implies *C* is optimal. □

## Data Availability

No new data were created or analyzed in this study.
